# New Insights on Solvent Implications in the Design of Materials Based on Cellulose Derivatives Using Experimental and Theoretical Approaches

**DOI:** 10.3390/ma14216627

**Published:** 2021-11-03

**Authors:** Anca Filimon, Mihaela-Dorina Onofrei

**Affiliations:** Polycondensation and Thermostable Polymers Department, “Petru Poni” Institute of Macromolecular Chemistry, Grigore Ghica Voda Alley 41A, 700487 Iasi, Romania; mihaela.onofrei@icmpp.ro

**Keywords:** (hydroxypropyl)methyl cellulose, poly(vinylpyrrolidone), solubility–viscosity correlation, thermodynamics, performance applications

## Abstract

The current paper presents a strategic way to design and develop materials with properties adapted for various applications from biomedicine to environmental applications. In this context, blends of (hydroxypropyl)methyl cellulose (HPMC) and poly(vinylpyrrolidone) (PVP) were obtained to create new materials that can modulate the membrane properties in various fields. Thus, to explore the possibility of using the HPMC/PVP system in practical applications, the solubility parameters in various solvents were initially evaluated using experimental and theoretical approaches. In this frame, the study is aimed at presenting the background and steps of preliminary studies to validate the blends behavior for targeted application before being designed. Subsequently, the analysis of the behavior in aqueous dilute solution of HPMC/PVP blend offers information about the conformational modifications and interactions manifested in system depending on the structural characteristics of polymers (hydrophilicity, flexibility), polymer mixtures composition, and used solvent. Given this background, based on experimental and theoretical studies, knowledge of hydrodynamic parameters and analysis of the optimal compositions of polymer mixtures are essential for establishing the behavior of obtained materials and validation for most suitable applications. Additionally, to guarantee the quality and functionality of these composite materials in the targeted applications, e.g., biomedical or environmental, the choice of a suitable solvent played an important role.

## 1. Introduction 

In recent years, a great interest was given to the research on the design of new polymeric materials with adapted properties and multiple functionalities, from technology to biomedical and environmental applications [1,2,3]. In this context, the physical mixing of two polymers or more represents simplest conceptual way to achieve the desired properties of new materials. However, due to the wide range of new applications, polymers from mixture must meet certain requirements considering their properties and interval/limit of miscibility [4,5,6,7]. Thermodynamic origin of phase behavior is well known and has a decisive influence on the polymer’s properties. Flory [8] concluded that “the immiscibility of two polymers may be taken as a rule, and miscibility as an exception”. In this regard, the formation of miscible mixtures is thermodynamically favored if the Gibbs free energy of mixing is less than zero [9]. This supposes the presence of some specific effects or interactions between the two components and such an interaction can dominate the properties of a system [4,6]. In other words, the thermodynamic interactions between the components of a mixture are the defining criteria for the characteristics of any polymer mixture. Thus, to obtain a proper miscibility, good compatibility and a thermodynamic equilibrium of polymer mixtures are necessary [7].

The study of polymer solutions is important to gain insight into practical processes (i.e., biological and industrial) by evaluating and controlling the flow properties and interactions study, as well as miscibility of polymers in a common solvent [8]. To anticipate the properties of the material and their interactions, on the one hand, the solubility parameters are usually used successfully [10]. Thus, the calculation of solubility parameters by the thermodynamic methods and understanding of the dissolution process allow the selection of a suitable solvent, as well as the optimization of design and processing conditions useful in membrane science, for example. On the other hand, various theories were formulated to allow the establishment of the solution properties on different concentration regimes [11,12]. To eliminate the inconveniences or complications that may occur in the determination and interpretation of intrinsic viscosity, various empirical equations were used [13,14,15], both in the case of neutral polymers and polyelectrolytes, in organic solvents, and water/organic solvent mixtures [6,7,16,17,18,19,20]. From this perspective, using the polymer blend concept, the present study focused on the examination of the properties in solution to gain necessary information for future processing and for further use of materials based on cellulose derivatives, i.e., (hydroxypropyl)methyl cellulose blended to hydrophilic synthetic polymer, poly(vinylpyrrolidone), for the specific applications in environmental and biomedicine. 

Nowadays, cellulose derivatives gained substantial consideration, due to their specific properties, e.g., biodegradability, biocompatibility, nontoxicity, solubility in water, etc., having large applicability in the chemical industries, biomedical, pharmaceutical, cosmetics, water desalination [1,2,21,22], as an effect of special viscometric behavior in solution. Particular properties of these derivatives can be optimized by utilization of polymer blends principle, which permits attaining of new models of materials, with a wide range of qualities [23]. In this context, (hydroxypropyl)methyl cellulose, a polar and hydrophilic polymer, is often used for designing a variety of materials with special properties, like solubility, hydrophilicity, and biocompatibility [24,25]. On the other hand, poly(vinylpyrrolidone) is one of the most investigated water-soluble polymers with special chemical and physical properties suitable to provide new materials with many applications in technology [3,26]. Due to the versatile properties, such as good solubility in water and organic solvents, nontoxicity, good hydrophilicity, biocompatibility, and polarity, poly(vinylpyrrolidone) can be used in pharmaceuticals, cosmetics, adhesives, pigments, coating electronics, bioengineering, textile materials, and water desalination [3,6,22,24,26]. It is well known that water is the most used solvent preferred by nature, due to its environmental compatibility, the low cost, nontoxicity, and nonflammable character. Besides, the use of water as a solvent for HPMC and PVP can influence their flow behavior, and hence, the finite product performance [27]. Taking into account all the above, in the present study, (hydroxypropyl)methyl cellulose and poly(vinylpyrrolidone) were selected due to their excellent physical properties, including their solubility in water, absence of toxicity, high hydrophilicity, flexibility, film-forming, and outstanding chemical and thermal stabilities [1,2,3,15,18,21,23,28]. Based on such versatile properties, an extensive attention was accorded to the use of both polymers as multifunctional materials for a variety of applications. This article addresses the particular need to prediction of solubility parameter of studied polymeric system to select the most suitable solvent, but also for prediction of physical properties, and implicitly, system performance. Therefore, the aim of this study is to analyze the behavior in aqueous dilute solution of HPMC/PVP polymeric system for acquiring information about the conformational modifications and interactions manifested in a common solvent, water, at different compositions of HPMC or PVP. Additionally, the particular details on the competition between varieties of interactions from multicomponent system were obtained by viscometric investigations in accordance with the evaluated hydrodynamic and thermodynamic parameters. All these aspects are essential for the design of multicomponent materials with targeted properties and establishment of the most appropriate compositions of this polymer blend for performance applications.

## 2. Experimental

### 2.1. Materials 

**Scheme 1:**
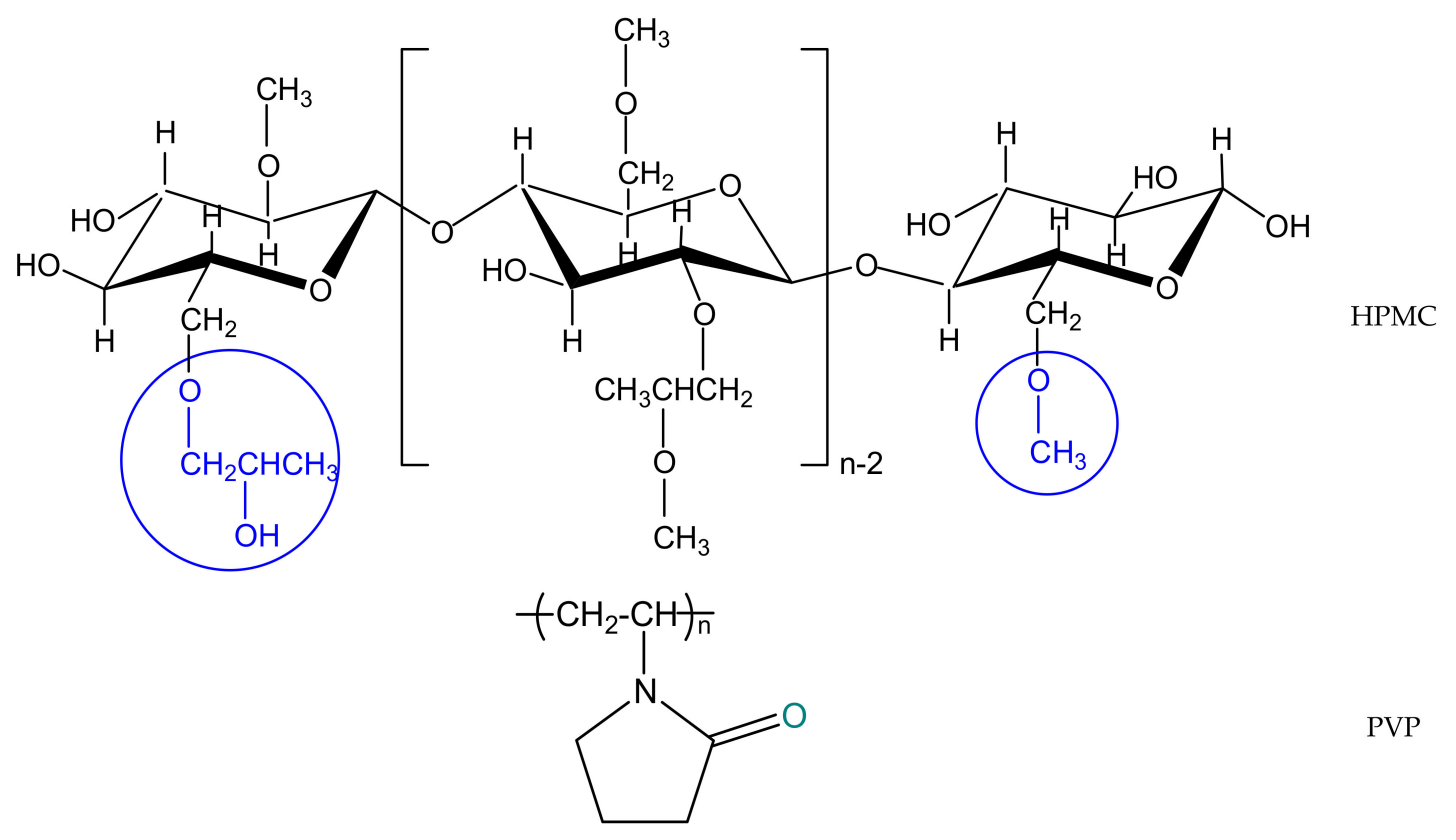
General chemical structures of investigated polymers.

(Hydroxypropyl)methyl cellulose (HPMC, Sigma-Aldrich Company, Darmstadt, Germany) presents a viscosity of 40–60 cP, 2% in water (20 °C). This polymer has a substitution degree for methoxyl and hydroxypropyl groups of 29% and 9%, respectively. Poly(vinylpyrrolidone) powder (PVP) with a molecular weight of 10,000 g/mol was also purchased from the Sigma–Aldrich Company (Darmstadt, Germany) and used as such. Solvents investigated in this work in the theoretical approaches, namely methanol (Me), acetic acid (AA), N,N—dimethylformamide (DMF), N-methyl—2—pyrrolidone (NMP) were purchased from Sigma–Aldrich (99.8% high purity, Germany), and the experimental study was performed in double distilled water (W). The chemical structures of the studied polymers, HPMC and PVP, are illustrated in Scheme 1. 

### 2.2. Preparation of the Polymer Mixture Solutions

To obtain polymers mixtures used in the experimental study, the homogeneous solutions of HPMC and PVP with known concentrations, *c*, varying between 0.01 and 0.5 g/dL were prepared by dissolution in double distilled water. Subsequently, obtained solutions were maintained for 24 h to reach equilibrium. By adjusting the volume ratios of the two stock homogeneous solutions, HPMC/PVP binary mixtures solutions in different mixing ratios, i.e., 100/0, 75/25, 50/50, 25/75, and 0/100 (*v*/*v*), were obtained.

### 2.3. Measurements

Viscosity measurements of the HPMC, PVP, and their blends were carried out in double distilled water in the 20–55 °C temperature range (±0.01 °C). The experiment was performed using a Schott viscometer AVS 350 with an Ubbelohde suspended-level viscometer, and the flow times data were obtained for various measurements with an accuracy of ±0.035%. Drainage errors caused by the viscometer used with a volume over 5 mL were insignificant and the corrections of the kinetic energy proved to be negligible.

Intrinsic viscosities, $\left[\eta \right]$, were determined applying Huggins [29] (Equation (1)) and Rao equations [14] (Equation (2)):

(1) $$\frac{\eta_{sp}}{c}=\left[\eta \right]+k_{H}{\left[\eta \right]}_{H}^{2}\cdot c$$

(2) $$\frac{1}{2\left(\eta_{rel}^{1/2}-1\right)}=\frac{1}{{\left[\eta \right]}_{R}\cdot c}-\frac{\left(a-1\right)}{2.5}$$

where the parameters involved are: $\eta_{sp}$-specific viscosity, $k_{H}$-Huggins constant, c is concentration of polymer solution, $a=1/\Phi_{m}$, and $\Phi_{m}$ is maximum volume fraction to which the particles can pack, expressed by $\Phi_{m}=\frac{\left[\eta \right]}{2.5}\cdot c_{m}$.

Starting from the phenomenological thermodynamics, using the generalized intrinsic viscosity, {η}, defined by Wolf [30,31], a better understanding of the flow behavior of polymer solutions over a wide of concentrations domain was allowed. In conditions of infinite dilution, $\left[\eta \right]$ reflects the specific hydrodynamic volume of the polymer coil that is surrounded by solvent and, at a given temperature, it is influenced by the solvent quality. Therefore, this new alternative method (Equation (3)) was used for the determination of the intrinsic viscosity and information on thermodynamic aspects by evaluating the hydrodynamic interactions parameters.

(3) $$\ln\eta_{rel}=\frac{c{\left[\eta \right]}_{W}+Bc^{2}{\left[\eta \right]}_{W}{\left[\eta \right]}^{•}}{1+Bc{\left[\eta \right]}_{W}}$$

In this equation the $\eta_{rel}$ represents relative viscosity, B represents hydrodynamic interaction parameter, and ${\left[\eta \right]}^{•}$ is the specific characteristic hydrodynamic volume.

The solubility of the polymers studied in different solvents was evaluated from theoretical approximations by applying the group contribution methods of Bicerano [32] and Stefanis [33].

## 3. Results and Discussion

### 3.1. Theoretical Background: Approaches to the Group Contributions to Calculate Solubility Parameters

Generally, the dissolution of substances in solvents is related to some thermodynamic properties of the dissolution process which have great significance to the study of solution structure. The solubility parameter represents an important factor in the solutions theory [34] and was shown the ability to be correlated to other physical properties [35]. Owing to the extensive use of polymer blends in technological advances, considerable efforts were made to establish the governing rules for the design of these mixtures. Thus, the solubility parameters of polymers and/or solvents are of major importance in the processing of polymer solutions for future industrial and technological applications [36,37]. From this reason, the solubility parameters estimation can be a useful tool to predicting the physical properties and performance of studied systems. Moreover, as noted in literature [38,39], application of polymers in industrial fields is critically dependent on the solubility parameter. In the literature of the field, various theoretical and experimental approaches were developed to evaluate the molecular affinities, solubility, and solubility bound phenomena. The principle “like dissolves like” may have a more general name, like “like seeks like”, used in the chemical sciences [40]. Such an approach, simple but with great impact, allows the quantitative evaluation of a system rendered by a numerical value assigned to the solubility parameter that reflects the molecular similarity or dissimilarity. The concept of solubility parameter is based on solubility theories that are linked to theory of nonideal solutions [40]. Based on these theories, for the occurrence of the solubility of two liquids, it is necessary that the intermolecular interactions between the molecules of the components the same type (A–A and B–B) are of the same order of magnitude and can be broken to form A–B interactions [41]. Because the molecules consist of structural fragments or groups with the ability to achieve such molecular forces and volume, the group contribution methods are used to evaluate solubility parameters considering only on the chemical structure. A variety of systems were studied from this perspective [39,40,42,43,44]. Their description by alternative methods can be laboriously and long lasting, reason why calculations of the values by group contributions method were extensively investigated. The application of such predictive methods, especially in the absence of experimental data, has special importance not only for selecting the appropriate solvents for each polymer from mixture, implicitly thermodynamic properties of the dissolution process, but also for the prediction of compounds properties, which allow the subsequent selection of compounds with desired properties for practical applications.

#### 3.1.1. Bicerano Formalism

To evaluate the Hildebrand solubility parameter of the polymers, δ, defined as the square root of its cohesive energy density, the theoretical study based on the group contributions method of Fedors and van Krevelen–Hoftyzer, according to the Bicerano formalism (Equation (4)) [32], was applied.

(4) $$\delta (298K)\equiv {\left[E_{coh}/V(298K)\right]}^{1/2}$$

This theoretical approximation implies the determination of the atomic and connectivity indices and comprises several steps:

Calculation of the zero-order connectivity indices, χ0 and χv0, and of the first-order connectivity indices, χ1 and χν1, according to the Equations (5)–(10).

(5) χ0≡∑1/δ

(6) χv0≡∑1/δv

(7) χ1≡∑1/β

(8) χv1≡∑1/βv

(9) $$\beta_{ij}\equiv \delta_{i}\cdot \delta_{j}$$

(10) $$\beta_{ij}^{v}\equiv \delta_{i}^{v}\cdot \delta_{j}^{v}$$

Table 1 presents the values of connectivity indices, δ, and $\delta^{v}$, used in the calculations.

Calculation of cohesive energy applying the group contributions of Fedors:

(11) Ecoh≈9882.5 ⋅ χ1+358.7⋅(6⋅Natomic+5⋅Ngroup)

where the involved parameters $N_{atomic}$ and $N_{group}$ are defined by following equations:

(12) $$\begin{array}{ll} N_{atomic}\equiv & 4\cdot N_{\left(–S–\right)}+12\cdot N_{sulfone}-N_{F}+3\cdot N_{Cl}+\\ & +5\cdot N_{Br}+7\cdot N_{cyanide} \end{array}$$

(13) $$\begin{array}{ll} N_{group}\equiv & 4\cdot N_{hydroxyl}+12\cdot N_{amide}+2\cdot N_{\left(nonamide-(NH)-unit\right)}+\\ & +4\cdot N_{\left(nonamide–(C=O)–next\;to\;nitrogen\right)}+\\ & +7\cdot N_{\left(–(C=O)–in\;carboxylic\;acid,\;ketone\;or\;aldehyde\right)}+\\ & +2\cdot N_{\left(other–(C=O)–\right)}-N_{\left(alkyl\;ether–O–\right)}-N_{C=C}+\\ & +4\cdot N_{\left(nitrogen\;atoms\;in\;six-membered\;aromatic\;rings\right)} \end{array}$$

The terms from Equation (12) are defined as: $N_{\left(–S–\right)}$—the number of sulfur atoms in the lowest (divalent) oxidation state; $N_{sulfone}$ number of sulfur atoms in the highest oxidation state (e.g., −SO_2_); $N_{F}$—numbers of fluorine atoms; $N_{Cl}$—numbers of chlorine atoms; $N_{Br}$—numbers of bromine atoms; $N_{cyanide}$—number of –C≡N groups.

In Equation (13):$N_{hydroxyl}$—number of –OH; $N_{amide}$—number of amide groups; $N_{\left(nonamide–(NH)–unit\right)}$—number of NH units from the nonamide structure; $N_{\left(nonamide–(C=O)–next\;to\;nitrogen\right)}$—number of C = O units from the nonamide structure next to nitrogen; $N_{\left(–(C=O)–in\;carboxylic\;acid,\;ketone\;or\;aldehyde\right)}$—number of C = O groups in carboxylic acid, ketone or aldehyde structures; $N_{\left(other–(C=O)–\right)}$—number of other C = O groups; $N_{\left(alkyl\;ether–O–\right)}$—number of alkyl ether–O–groups, $N_{C=C}$—number of carbon-carbon double bonds, excluding any such bonds found along the edges of the rings; $N_{(\mathrm{nitrogen}\;atoms\;in\;six-membered\;aromatic\;rings)}$—number of nitrogen atoms in six-membered aromatic rings.

Calculation of the molar volume:

(14) V(298K)≈3.642770⋅ χ0+9.798697⋅ χv0−8.542819⋅ χ1++21.693912⋅ χv1+0.978655⋅NMV

(15) $$\begin{array}{ll} N_{MV}\equiv & 24\cdot N_{Si}-18\cdot N_{\left(–S–\right)}-5\cdot N_{sulfone}-7\cdot N_{Cl}-16\cdot N_{Br}+\\ & +2\cdot N_{(backbone\;ester)}+3\cdot N_{ether}+5\cdot N_{carbonate}+\\ & +5\cdot N_{C=C}-11\cdot N_{cyc}-7\cdot (N_{fused}-1) \end{array}$$

(last term only to be used if $N_{fused}\geq 2$)

where: $N_{Si}$—number of silicon atoms; $N_{\left(backbone\;ester\right)}$—number of ester (–COO–) groups in the backbone of the repeating units; $N_{ether}$—total number of ether (–O–) linkages in the polymeric repeating unit. Note that only the (–O–) linkages between two carbon atoms will be counted as ether linkages in $N_{ether}$; $N_{carbonate}$—number of carbonate (–OCOO–) groups; $N_{cyc}$—number of nonaromatic rings (i.e., “cyclic” structures) with no double bonds along any of the ring edges; $N_{fused}$—number of rings in fused ring structures.

Hildebrand solubility parameter defined by Equation (4) it is applied only for regular solutions, namely solutions in which the polar and/or specific interactions between molecules are neglected. Due to this limitation, approach developed by Hansen [45,46] is the widely accepted. Therefore, so—called Hansen solubility parameter is extension of the Hildebrand solubility parameter for the systems in which polar and hydrogen bonding are present.

#### 3.1.2. Prediction of Hansen Solubility Parameters: A New Group-Contribution Method

Over time, the research undertaken in the study of the solubility parameter has evolved and introduced new hypothesis to overcome inconsistencies of the Hildebrand solubility parameter. Burrell [47] introduces the hypothesis, according to which, the highest solubility occurs between materials with similar polarities. This premise leads to the division of solvents into three categories taking into account the hydrogen bond. Subsequently, Hansen [41,45,46] introduces new hypotheses according to which the cohesive energy that defines the solubility parameter has three components corresponding to specific interactions. Thus, the first type of interaction is the nonpolar one; it occurs as a result of the orbit of the charged electrons, belonging to each atom, around a central nucleus, positively charged. The moving negative charges generate an electromagnetic field that attracts all atoms to each other, regardless of direction [48]. All molecules exhibit this type of attraction force. The second type of interaction, defined by Hansen as polar interactions, is generated by permanent dipole-dipole interactions and contributes to the dipole moment of the molecule [38]. Most molecules have these types of inherent molecular interactions. Hydrogen bonding is third type of interaction. These bonds are considerably weaker than covalent bonds but are much stronger than the dipole–dipole interactions. Therefore, the application of such a predictive method in evaluation of solubility parameter that takes into account all three components corresponding to specific interactions, namely three–dimensional solubility parameters or the Hansen solubility parameters, is much more appropriate than the Hildebrand solubility parameter. For this reason, the group contributions method was extended by Stefanis et al. [33] to predict the Hansen solubility parameters. Thus, the total solubility parameter, $\delta_{t}$, expressed as sum of the square root of the Hansen solubility parameters [45,49], is defined according to Equation (16).

(16) $$\delta_{t}={\left(\delta_{d}^{2}+\delta_{p}^{2}+\delta_{H}^{2}\right)}^{1/2}$$

In this new method of the group contributions, the molecular structure of studied compounds can be described using two types of functional groups, namely first-order groups, which correspond to the basic molecular structure of compounds [50] and second–order groups, which have the first-order groups as building blocks. According to these statements, equation which gives the values of three-dimensional solubility parameters ($\delta_{d}$, $\delta_{p}$, $\delta_{H}$) is follows:

(17) $$\delta ={\left(\sum_{i}N_{i}C_{i}+W\sum_{j}M_{j}D_{j}\right)}^{}$$

where $C_{i}$ represents the first-order contribution of structural group of type i that appears of $N_{i}$ times in the studied compound and $D_{j}$ is the second–order contribution of structural group of type j that appears of $M_{j}$ times in that compound, and W is a constant, that gets equal to zero for compounds without second-order groups and equal to 1 for compounds with second-order groups.

According to the latter corrections, the new updated method of the group contributions defines the Hansen solubility parameters by following equations (Equations (18)–(20)) [39,51]:

(18) $$\delta_{d}={\left(\sum_{i}N_{i}C_{i}+\sum_{j}M_{j}D_{j}+959.11\right)}^{0.4126}$$

(19) $$\delta_{p}=\left(\sum_{i}N_{i}C_{i}+\sum_{j}M_{i}D_{j}+7.6136\right)$$

(20) $$\delta_{H}={\left(\sum_{i}N_{i}C_{i}+\sum_{j}M_{j}D_{j}+7.7003\right)}^{}$$

According to above-mentioned, for choosing the most suitable solvents, the use of Hansen solubility parameters is much more proper than the Hildebrand solubility parameter. Additionally, the solubility behavior established by only the Hildebrand solubility parameter because the solubility properties can be affected by any type of specific interactions, especially hydrogen bonds, crosslinking, temperature, and changes in temperature, solvent molecules size, and shape.

Given this background, several graphing and modeling techniques were developed to aid in the prediction of solubility behavior [52,53]. For example, a modeling technique of three–dimensional solubility was developed by Hansen [41,54], which considered that the total solubility parameter of a polymer represents a point located in three–dimensional space. This point is the center of a sphere, so-called Hansen solubility sphere, with radius $R_{0}$, named the interaction radius of that polymer defined by Equation (21).

(21) $$R_{0}={\left(\delta_{d}^{2}+\delta_{p}^{2}\right)}^{1/2}-\delta_{H}$$

The distance between the total solubility parameters of the solvent and polymer, respectively, also named the solubility parameter distance, $R_{a}$, is given by the following equation (Equation (22)) [35,55,56,57]:

(22) $$R_{a}^{2}={\left(\delta_{d,p}-\delta_{d,s}\right)}^{2}+{\left(\delta_{p,p}-\delta_{p,s}\right)}^{2}+{\left(\delta_{H,p}-\delta_{H,s}\right)}^{2}$$

where $\delta_{d}$, $\delta_{p}$, and $\delta_{H}$ are the Hansen solubility and the “*p*” and “*s*” terms correspond to the parameters of the polymer and solvent, respectively.

Also, another parameter used in the description of the solubility behavior, implicitly the solvent quality, is the relative energy difference, $RED=R_{a}/R_{0}$. According with literature [35], when value of *RED* is lower than unity, it corresponds to good solvents, while as the quality of the solvent progressively decreases, the *RED* value increases.

Overall, the solubility parameter concept was initially applied to liquid mixtures, subsequently, has expanded to solid-liquid systems being notable for its great utility in various e.g., coating industry, cosmetics, pharmacy, and biology [10,36,58]. For this reason, as above mentioned, the development of such predictive methods is importance not only for the choice of suitable solvents for each given polymer but also evaluation of the thermodynamic and hydrodynamic properties of the solution with impact on the subsequent selection of compounds with special properties for diverse practical applications.

### 3.2. Thermodynamic Approach Based on Group Contribution Methods to Evaluate the Solubility Parameters of HPMC/PVP/Water System

In most applications, the miscibility/solubility of polymers in a particular solvent is one of the basic requirements to knowledge, understand, and explain the physico-chemical properties, and also for establishing the most appropriate compositions of the polymer blends with desired properties for specific applications [59,60]. Based on literature studies mentioned above [32,38,39,59,60,61,62], in this research, the different theoretical models based on the group contributions were utilized for to estimate the solubility parameters, to predicting systems physical properties and implicitly, system performance.

As an initial attempt to explore the behavior of the system contenting (hydroxypropyl)methyl cellulose and poly(vinylpyrrolidone) in practical applications, the solubility parameters in various solvents were evaluated. As is well known many parameters can compete, however, the solubility parameters of polymers are extremely important in modeling, simulating, and optimizing of the studied systems in biomedical and industrial applications. In this sense, in the first stage, the solubility parameters were calculated, according to Bicerano formalism, using values of zero—(χ0 and χv0) and first-order connectivity indices (χ1 and χν1), evaluated by Equations (5)–(10). Table 2 presents the values of these parameters calculated with the atomic simple connectivity indices, δ, and of the valence connectivity indices, $\delta^{\nu }$, from literature [32] (see Table 1), according to the contributions of each structural group from the polymer chains of HPMC and PVP.

Solubility parameters theoretical calculated (Equation (4)) for HPMC and PVP with cohesive energy, $E_{coh}$ (Equations (11)–(13)) and molar volume, V, (Equations (14) and (15)) are presented in Table 3.

Subsequently, for a better understanding the properties of studied polymers and their interactions, the study was extended using another thermodynamic model to correlated of the measured solubility values. Thus, the work attempts to enhance the capacity of solubility parameters by applied a new method over the classical approach, incorporating into their evaluation the other basic rule of solubility, namely, the rule of “complementarity matching” [51]. In this sense, the Hansen solubility parameters (Equation (16)) for HPMC and PVP were evaluated by application of updated method by computer-aided molecular design (Equations (17)–(20)) [51] using the first- and second-order groups contributions to the dispersion partial solubility parameter, $\delta_{d}$, the polar partial solubility parameter, $\delta_{p}$, and the hydrogen-bonding partial solubility parameter, $\delta_{H}$, according to Table 4. Total solubility parameters of HPMC and PVP using contributions of the dispersion, polar, and hydrogen bonding of the partial solubility parameters were calculated and listed in Table 5, taking into account the degree of substitution in the case of HPMC. Also, in Table 5 are listed solubility parameters values for different solvents [63], to establish their quality on the studied HPMC/PVP system.

The solvent selection is often empirical and is usually driven by the need to find a common solvent or solvents mixture for all system components. However, the features of the solvent can influence the properties of the resulting system differently. Generally, the solubility behavior of a polymer is influenced by the polymer structure and also by interactions of polymer–solvent [64]. According to the obtained values of solubility parameters from Table 5, the contribution of the interactions may occur in the system polymer-solvent. Thus, the dispersive forces (i.e., van der Waals interactions) are approximatively similar for the HPMC and PVP, while the forces between molecules of permanent dipoles (polar contribution) and hydrogen bonding components are extremely different. Therefore, this specific energy contribution, reflected by the presence of significant intermolecular interactions between various molecules in the system, has a strong effect on the values of partial solubility parameters of the two polymers. Therefore, the presence of significant intermolecular interactions between various molecules in the system explain the values of partial solubility parameters of the two polymers.

**Figure 1:**
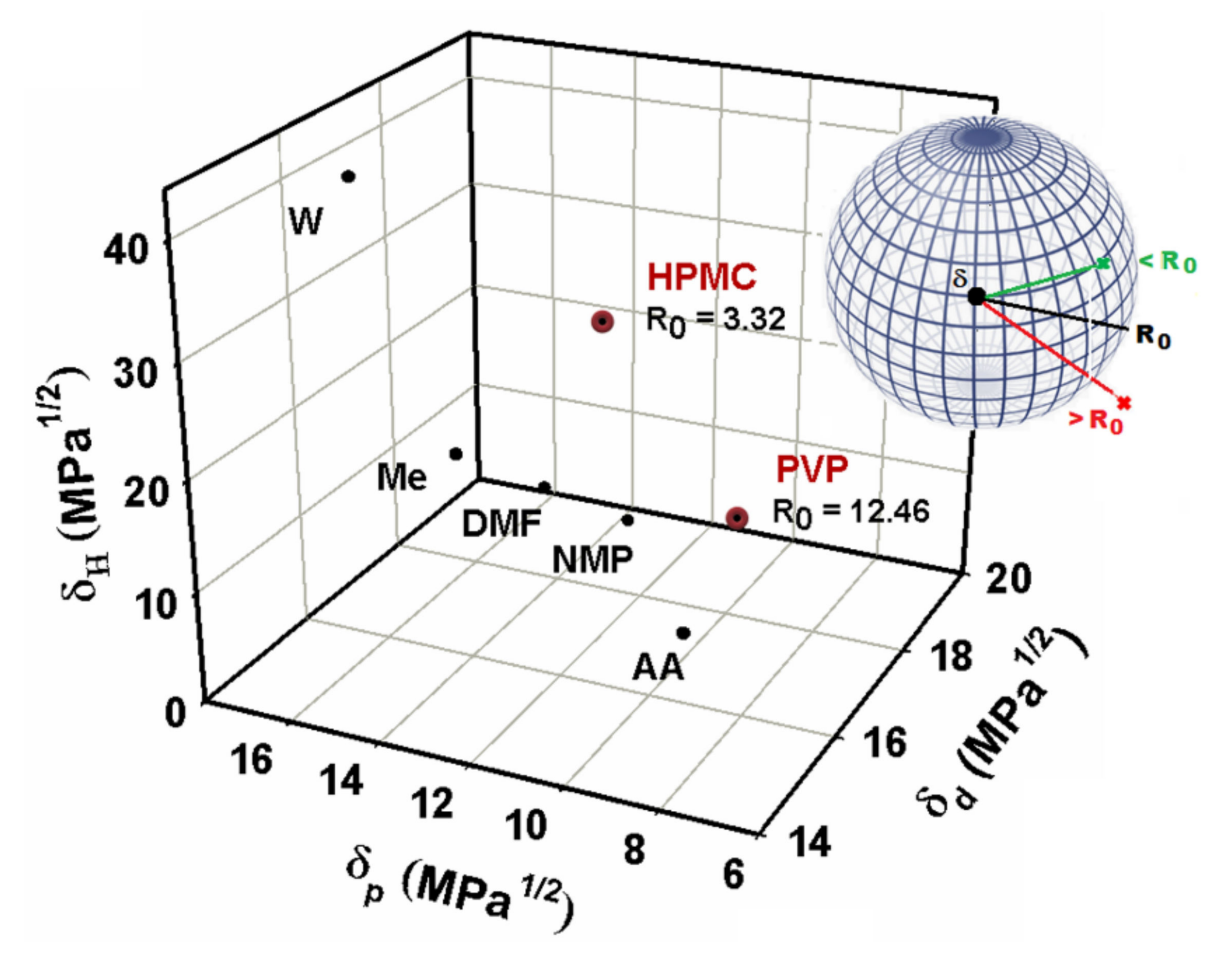
3D representation used to plot solubility information for studied polymers with interaction radius, $R_{0}$, compared to that of different solvents situated in same compatibility region. A schematic illustration of Hansen’s solubility sphere theory.

Hence, significantly differences between the polar and hydrogen bonding parameters of HPMC and PVP were obtained, because the HPMC is a polar polymer with hydrogen bonding ability. This fact indicating a strong tendency of HPMC components to interact in aqueous conditions, and therefore, a good affinity polymer–solvent [39,62]. On the other hand, the correlation of the polymer’s solubility data with the Hansen parameters of the used solvents can be viewed in Figure 1, where the Hansen parameters values are represented in three-dimensional graphic. Practically, the use of Hansen parameters is based on the principle “like dissolves like” and depending on the parameters of a given solvent, one can predict their ability to solvate the polymers. Thus, according to the parameters obtained by the Hansen theory (see Table 6) was possible to explain the relationship between the solubility parameters with all the solvents situated in the same region, called “compatibility region”. Moreover, we investigated the role of water, extending the study to evaluate the solvent characteristics and conformation of the polymers with respect to the phase behavior of the HPMC/PVP system.

Generally, the strength of the interactions between solute and solvent can be reflected by the polarity, as well as the capacity of hydrogen bond donor and acceptor of solvents [65,66]. In this sense, findings from this study indicate that water is a better solvent for HPMC compared to PVP, deduced statement from the Hansen theory that predicts that for compounds with different molecular weight but with the same behavior in aqueous solution on their ability to generate the hydrogen bonds interactions, the radius of the sphere decreases when the molecular weight increases (according to Figure 1) [67]. Therefore, for solvents that are distant from the center of the solubility sphere (border solvents), only the light fraction of the samples can be solubilized and then, the apparent solubility is low.

For solvents that are close to the center of the solubility sphere, the heavier fraction can also be solubilized, and then the apparent solubility is higher [55]. Another indicator derived from this study is based on the complexity of the water molecules, that are small and more polar (polarity index = 9; dipole moment = 1.855), and so can interact very easily with HPMC and PVP, conducting to the strong interactions between the components [39,68]. Consequently, as shown in Figure 1, the relationship between the solubility and polarity of the studied solvents group shows a good correlation, and the solubility increases with the increasing solvents polarity, suggesting that the dissolution of polymers in the polar solvents chosen is in accordance with the principle of “like dissolves like” [69]. Water has the highest value of the polarity index and the hydrogen bond donation propensity, which indicates that the hydrogen bond donation capacity of solvent plays a primary role in determining solubility [59]. Therefore, we deduced that there is hydrogen bonding among water and studied polymers (HPMC and PVP) molecules, the aspect that will be discussed later by the viscometric study. Based on these remarks, this approach is useful to provide insight into the miscibility of a polymer with a solvent. Thus, the results of solvation clearly indicate that intermolecular interactions between polymer and solvent represent one of the critical factors, determining the solubility of HPMC and PVP in polar solvents. In this research, the physico–chemical properties and their derived parameters provide important information concerning the nature and strength of intermolecular interactions in mixtures, so as to further understand conformational behavior of these mixtures in the dilute solution.

### 3.3. Viscometric Behavior of HPMC/PVP System in Water

**Figure 2:**
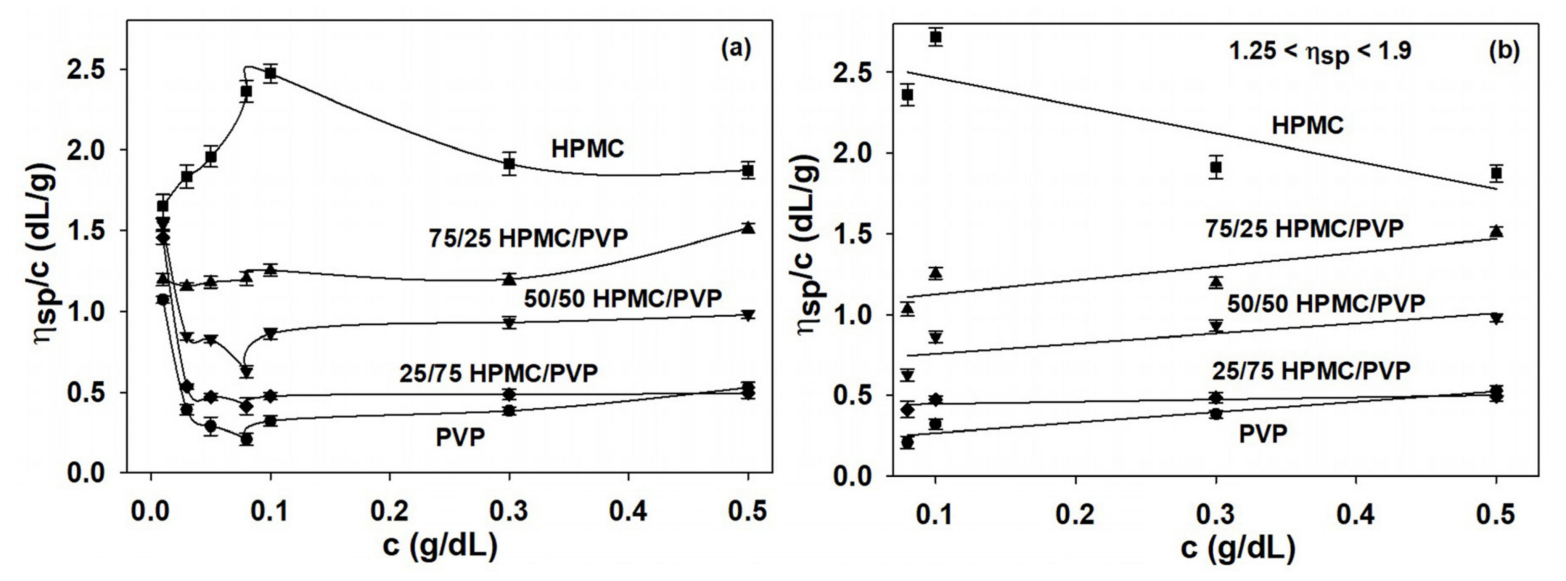
Huggins plots for HPMC, PVP, and HPMC/PVP mixture in water at different mixing ratios and 25 °C: (**a**) over extremely dilute/dilute concentration domain and (**b**) over the dilute domain, where condition $1.25<\eta_{rel}<1.9$ was respected.

Viscosity studies provide the most useful information about behavior of polymers in solution on the conformational changes, the flexibility, interactions between polymer chains or polymer-solvent, as well as the hydrodynamic parameters [5]. Overall, the increase in viscosity is due to the intermolecular interactions, while the decrease in viscosity is the result of intramolecular interactions [70,71]. Another factor responsible for the properties of the polymers is represented by the miscibility of the polymers from the system, their mixing ratios, and quality solvent. Thus, all these features influence the specific interactions established among the components of the complex polymeric systems and, hence, the viscometric behavior [70,72,73]. In the given context, by application of the Huggins equation (Equation (1)), Figure 2 offers indications on the conformation of polymer chains and balance between the forces which occur in complex system, HPMC/PVP/water.

Hence, by analysis of Huggins plots for pure components HPMC, PVP and their mixtures in water at different concentrations and compositions (Figure 2) were observed that, as the concentration decreases, upward changes in the slope of $\eta_{sp/c}$ as a function of concentration for HPMC occur. Moreover, in case of HPMC sample, the reduced viscosity presents a maximum value at a certain concentration and as the concentration decreases a rapid decrease occurs. For HPMC/PVP mixtures, the maximum value of reduced viscosity is less observed. According to these observations, a higher HPMC content in the polymer mixtures causes an increase of viscosity, which indicates an increase of coil dimension in solution. On the other hand, the interactions between PVP and HPMC in solution determine a deviation of the specific viscosity of the polymer mixture. Thus, the positive deviations indicate a better tendency for heterogeneous interactions among the PVP and HPMC macromolecules, than homogeneous interactions among macromolecules of the same polymer. In addition, the positive or negative deviations were assigned to the self-assembling phenomena or the development of interpolymer complexes from polymers in solutions. Furthermore, in the conditions of temperature, composition, and solvent the average dimensions of the macromolecules decrease when repulsive interactions occur between the two polymers [5]. The sudden variations that occur in the low concentration range are a consequence of the volume occupied by the polymer molecules which is smaller than the available volume and these are attributed to the conformational changes of the macromolecules [74].

The observed viscometric behavior can be explained by the increasing of hydrodynamic and thermodynamic interactions, and thus, mutual attraction of macromolecules in solution. Hence, HPMC/PVP/water mixture is considered to be miscible and indicates that exist interactions between HPMC and PVP in the form of intra- and intermolecular hydrogen bonding. According to the literature [72], HPMC and PVP present a similar behavior in water and each one of them looking for hydrogen bond donor. This fact demonstrated that the HPMC and PVP are miscible between them [75], according to previous results concerning the solubility behavior.

**Figure 3:**
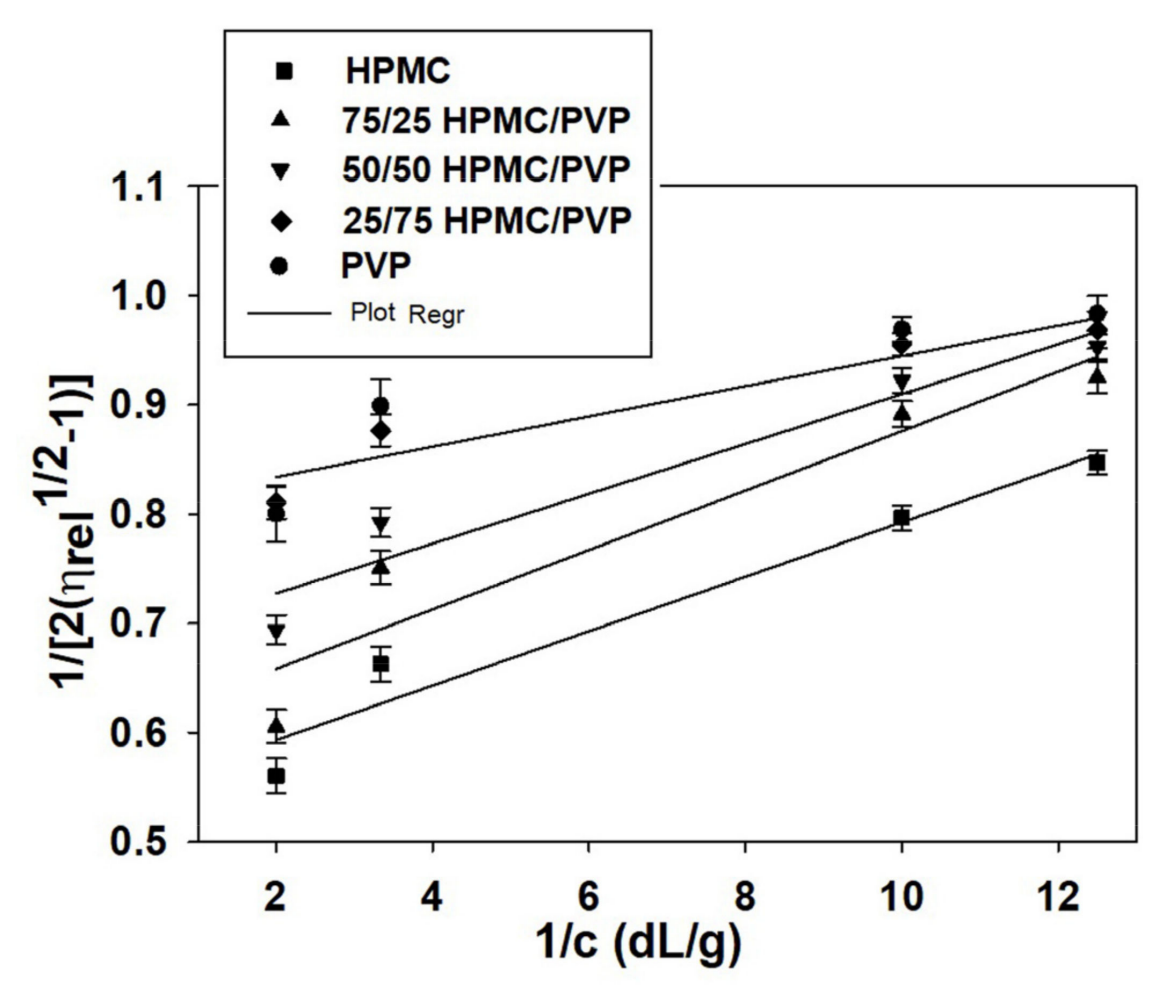
Rao plots for HPMC, PVP, and HPMC/PVP mixture in water at different mixing ratios and 25 °C.

**Figure 4:**
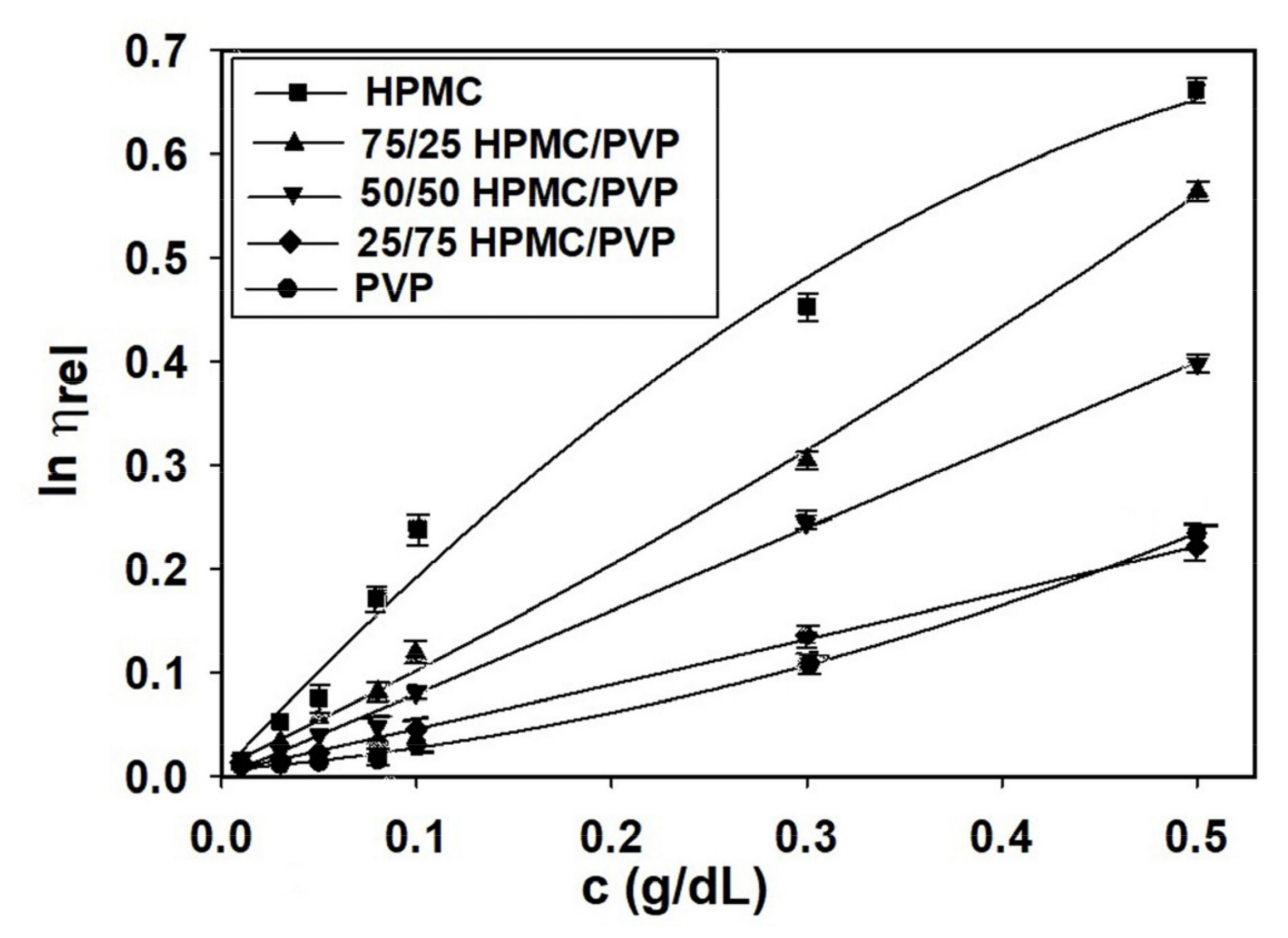
Plots of $ln\eta_{rel}$ as a function of concentration for HPMC/PVP/water at different mixing ratios and 25 °C.

Generally, at low polymer concentrations (extremely dilute domain), where the values of relative viscosity are lower than 1.2, the reduced viscosity shows deviations from the linear dependency (the Huggins equation, Figure 2b). This fact makes it difficult to determine the intrinsic viscosity through extrapolation to zero polymer concentration (see Figure 2a). However, it should be underlined that the irregularity of viscosity which occurs in the extremely dilute concentration domain is generated by the adsorption phenomena produced on the viscometer wall surface as a result of the functional groups of neutral polymers involved [70]. In this regard, the deviations from linearity reported in the extremely dilute concentration domain (Figure 2a) can be eliminated using different approximations, namely the Rao approximation [14]—slightly sensitive to the possible errors occurring in relative viscosity data (Figure 3), or Wolf approximation [17,18,19,20]—applicable to charged and/or uncharged polymer solutions at sufficiently low polymer solution concentrations (Figure 4).

Thus, for obtaining information concerning the hydrodynamic parameters and to eliminate these deviations from linearity for ternary system composed by HPMC/PVP/water, the more recent approach proposed by Wolf (Equation (3)) proved to be suitable to find out intrinsic viscosity for neutral polymers over a wide concentration domain [5]. So, by applying the Wolf equation (see Figure 4), the intrinsic viscosity, ${\left[\eta \right]}_{W}$, the ${\left[\eta \right]}^{•}$ and B parameters were evaluated and are presented in Table 7 together with viscosity parameters obtained by Huggins method.

From the obtained results, all calculated curves are in accordance with the experimental data and certify that Equation (3) is proper to evaluate the viscometer and hydrodynamic parameters. Moreover, it should be mentioned that values of intrinsic viscosity obtained by Wolf equation are in accord with those resulting from Huggins equation (Table 7). Additionally, data concerning behavior of PVP in solution are in agreement with results obtained in literature studies [5].

Also, the analysis of the experimental data revealed that for HPMC solution a high value of the intrinsic viscosity was obtained compared to that of PVP. In this context, according to the literature [76], the molecular weight and chain length have a considerable effect on the viscosity of HPMC aqueous solutions. On the other hand, the viscometric behavior of HPMC/PVP mixtures has evolved in a nonpredictible manner on whole composition range, as a result of the inter- and intrapolymer interactions between HPMC and PVP in aqueous solution, as well as hydrogen bonding or association phenomena occurring in a solution of polymer mixtures.

**Figure 5:**
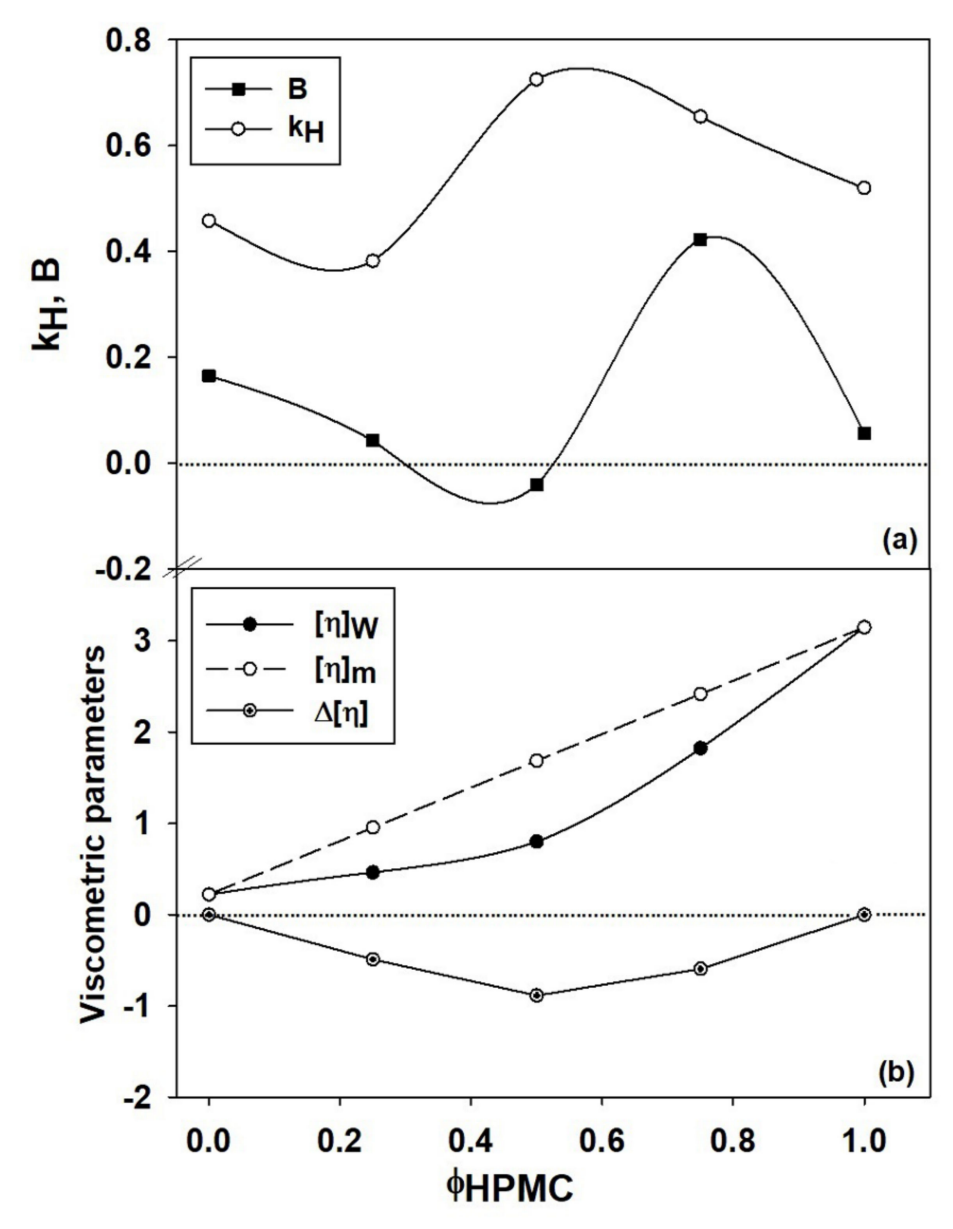
Dependence of $k_{H}$ constant, B parameter (**a**) and of viscometric parameters (${\left[\eta \right]}_{W}$, ${\left[\eta \right]}_{m}$, Δ[η]) (**b**) on the volume fraction of HPMC. Dotted line from (**b**) part indicates additive rule (${\left[\eta \right]}_{m}^{id}=\sum {\left[\eta \right]}_{i}\phi_{i}$).

To describe the viscometric behavior of HPMC/PVP system, two parameters (${\left[\eta \right]}_{W}$ and B) from Equation (3) are enough; the contribution of ${\left[\eta \right]}^{•}$ being zero [77,78]. As is known from the literature [77,79,80], ${\left[\eta \right]}^{•}$ is an adjustable parameter only required for the polyelectrolytes in salt free aqueous solutions, incorporating the effect of electrostatic interactions on hydrodynamic volume of charged macromolecules at finite concentration [17]. Thus, the hydrodynamic interaction parameter B, quantifies the interactions between the polymer segments belonging to different macromolecules, as well as those polymer-solvent. In the dependence of $\ln\eta_{rel}$ on concentration, presence of the curvature generated by these interactions, can be bent downwards, corresponding to B>0 or upwards when B<0 [74]. According to Wolf evaluation [17], for almost all uncharged polymers B has positive values, indicating the existence of favorable polymer-solvent interactions and takes negative values under unfavorable thermodynamic conditions. Figure 5a and Table 7 show that for the system investigated, the B parameter presents positive (as expected in the case of the neutral polymers) and negative values, suggesting presence of both polymer-solvent and polymer-polymer interactions. Thus, the negative value of B parameter at 50/50 (*v*/*v*) composition of HPMC/PVP blend, corresponding to the maximum value of $k_{H}$ validates the presence of the interactions between polymer chains which are predominant exceeding the contribution of polymer-solvent interactions. Moreover, the macromolecular aggregation phenomenon or hydrogen bonding formation can appear due to the strength of polymer-polymer interactions.

Also, the influence of the polymer–solvent interactions, which produce on the one hand, a decrease of $k_{H}$ below of 0.5 and positive B values for PVP sample and mixtures with higher content of PVP, and on the other hand, for HPMC sample generate of $k_{H}$ value close to 0.5, suggesting that water behaves as marginal solvent and both types of interactions are present in the system.

**Scheme 2:**
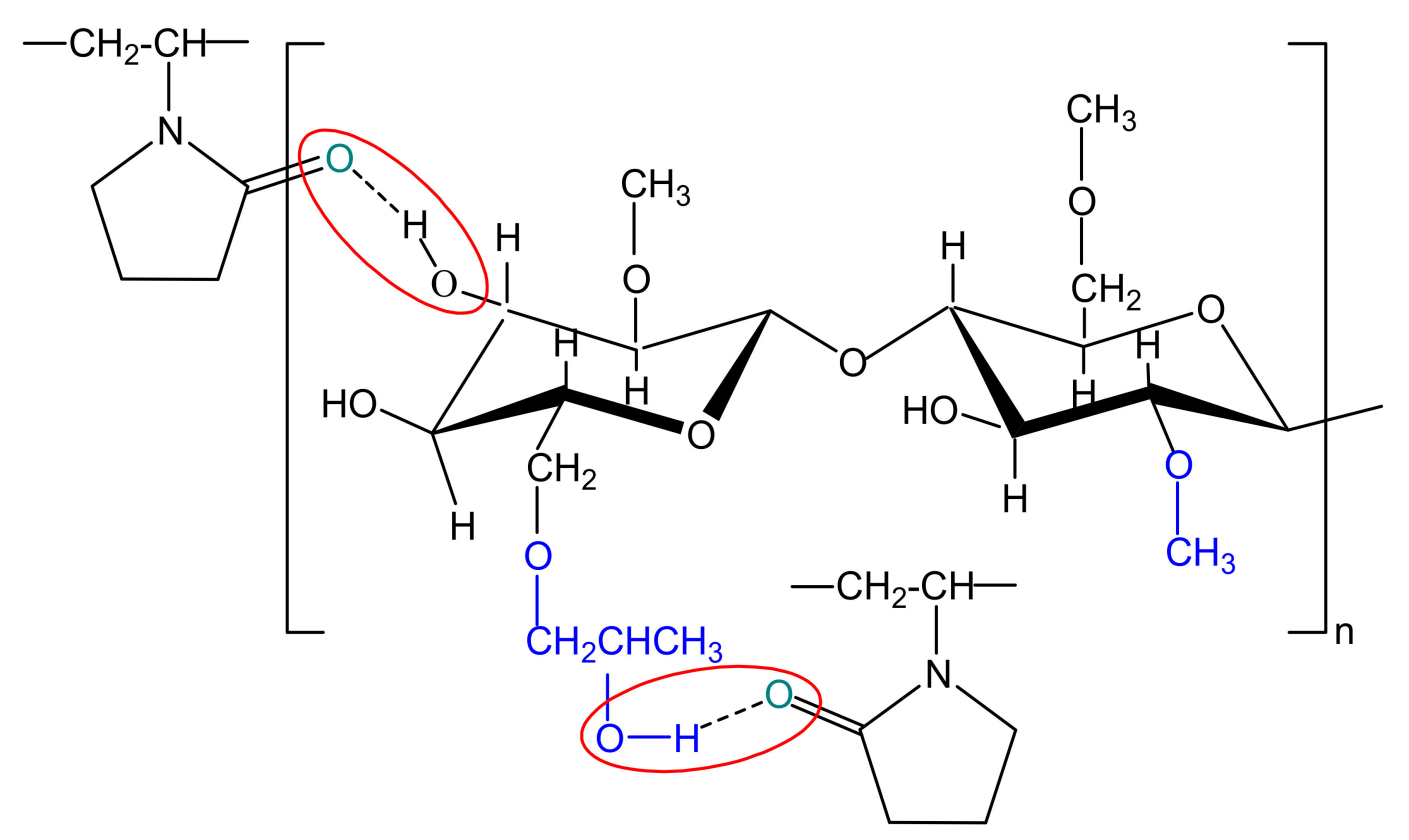
Hydrogen-bonding interactions which could appear between hydroxyl groups of HPMC and carbonyl groups of PVP.

For multicomponent systems, the dependence of intrinsic viscosity on composition is complex, being influenced both by conformational change of each type of polymer from blend and by the thermodynamic or hydrodynamic interactions from polymers (see Figure 5b). Deviation from the ideal value of intrinsic viscosity, given by the additive rule (${\left[\eta \right]}_{m}^{id}=\sum {\left[\eta \right]}_{i}\phi_{i}$, ${\left[\eta \right]}_{i}$ and $\phi_{i}$ represent the intrinsic viscosity and volume fraction of polymers, respectively (*i* = 1 refers to polymer (1), while *i* = 2 refers to polymer (2)), is attributed to the effects of the thermodynamic interactions manifested between the two polymers from solution [81,82], defining the miscibility state. However, the miscibility limit in dilute polymer solutions can be affected, on the one hand, because the intermolecular interactions are relatively rare and masked by solvent molecules, and on the other hand, the polymer–solvent interactions are not constant over the entire composition range [83]. According to literature [79], the positive or negative deviations from the additive rule were attributed to self–assembling phenomena, hydrogen–bonding or the formation of interpolymer complexes between fully miscible polymers in dilute solutions. Thus, based on the statements previously presented, according to Figure 5b, the dimension of the polymer coil gradually increases as the HPMC content increases and the polymers miscibility in dilute solution over the entire composition range it is the result of the competition among different thermodynamic interactions. Therefore, the increase of intrinsic viscosity can be attributed to the repulsive intermolecular interactions between HPMC and PVP which generate increases of the excluded volume effects. Additionally, in Figure 5b, a negative deviation is observed, given by $\Delta [\eta ]={\left[\eta \right]}_{W}-{\left[\eta \right]}_{m}^{id}$, suggesting that HPMC presence in higher amount in the polymer mixture negatively affects the polymer–solvent interactions and favors the polymer–polymer interactions. Moreover, according to the dependences shown in Figure 5b, addition of PVP to the solution of HPMC favors hydrogen-bonding interactions between hydroxyl groups of HPMC and carbonyl groups of PVP (see Scheme 2), contributing to the specific molecular rearrangement of the system. In this sense, some recent results obtained for HPMC/PVP solutions in water, mention that hydrogen bonding between the hydroxyl groups of HPMC and the carbonyl groups of PVP are highly intense when HPMC is added in the system [72]. In addition, HPMC and PVP present an opposing mechanism of hydrogen bonding, where HPMC act as a proton donor and PVP as a proton acceptor [84].

However, the viscometric behavior of HPMC/PVP system depends significantly on the polymer concentration and decrease as increasing of PVP content. According to literature, the polymer chains are separate and present a poorly interaction in dilute regime. Also, when the PVP is added to the HPMC aqueous solution, the system creates a complex by the hydrophobic interactions. This might be due to the nature of the PVP, which contains a strong hydrophilic moiety-pyrrolidone and a strong hydrophobic moiety-alkyl group. Consequently, the effect of the interaction of PVP and HPMC in water could affect the conformation of the chain due to the hydrophobic effect. Moreover, based on literature data, the polar groups present in the HPMC structure are capable to interact with different solvents, including water, by polar forces and intermolecular hydrogen bonds [23,85,86]. Hence, the influences of interactions between these polymer groups are responsible for the miscibility of polymers.

The conformational modifications in the polymer/polymer/solvent system are generated by the intermolecular hydrogen-bonding and by the hydrogen-bonding with water. Thus, the presence of solvent in the system leads to new types of interactions, which determine different conformations depending on the concentration and temperature [87]. Customarily, the capacity of polymers to form compatible mixtures implies the occurrence of favorable intermolecular interactions between the different polymer chains [88]. To improve viscosity characteristic is necessary that water behaves as a good solvent for both polymers used. Therefore, if the water is a good solvent for the polymers, the polymer-solvent interactions cause the solvent penetration into the macromolecular coils, an increase of size, as well as of viscosity. Thus, in particular, the results derived from solution measurements are consistent with those on the solvent quality (reflected by values of the solubility parameters), evidencing that HPMC and PVP form a thermodynamically miscible mixture. Consequently, the obtained results concerning the relationship between the solubility properties and structural—conformational features of these polymers prefigure the existence of some specific characteristics that recommend this polymer mixture for the targeted applications. Also, through the realized research were identified the optimal molecular aspects which represent the basis to future applications of this blend in the production of performance membranes with specific properties required for biomedical, environmental applications, as predicted.

## 4. Conclusions

This article focuses on the development of new materials designed for target applications from biomedical, pharmaceutical, or environmental fields. In this context, HPMC and PVP were selected for their excellent physical properties, including their solubility in water, absence of toxicity, high hydrophilicity, flexibility, etc. The dynamics study of solutions based on cellulose derivatives is imperative to obtain a proper control of the flow properties to establish possible applications. This wide variety of applications is a consequence of their particular behavior in solution. Thus, the system miscibility, as well as the choice of suitable solvent was discussed through the solubility parameters evaluated by experimental and theoretical approaches. Moreover, the viscometric data were interpreted by the Huggins and Wolf models as a function of polymers structural characteristics (i.e., hydrophilicity, flexibility), quality of solvent, and polymers mixture composition. Thus, from analysis of viscometric data, namely, the intrinsic viscosity, Huggins constant and, B parameter, and information concerning the competition between variety of interactions manifested in the HPMC/PVP/water ternary system were obtained. Additionally, viscosity dependence on polymers composition was affected by the conformational modifications of polymers compounds from mixture and by the hydrogen bonding.

The acquired information provides the possibility to predict the properties of analyzed materials and to optimize the design and processing conditions to obtain films, membranes, or fiber with targeted properties. Hence, by the research undertaken, this paper represents an innovative route for optimization of the materials characteristics to validated for most suitable applications.

## Figures and Tables

**Table 1:** *δ* and $\delta^{v}$ values used for calculation of zero—and first-order connectivity indices [32].

Atom	Hyb	$N_{H}$	δ	$\delta^{v}$
C	sp^3^	3	1	1
		2	2	2
		0	4	4
	sp^2^	1	2	3
		0	3	4
N	sp^3^	0	3	5
O	sp^3^	1	1	5
		0	2	6
	sp^2^	0	1	6

**Table 2:** Zero-order connectivity indices, χ0 and χv0, and first-order connectivity indices, χ1 and χv1 for HPMC and PVP.

Polymers	Connectivity Indices			
	χ0	χv0	χ1	χv1
HPMC	18.40	18.81	15.43	10.88
PVP	5.56	4.76	3.88	2.82

**Table 3:** Bicerano formalism parameters used in the calculation of HPMC and PVP solubility parameters.

Materials	Parameters		
	$E_{coh}\cdot 10^{4},\;(J/\mathrm{mol})$	*V* (298 K), (mL/mol)	δ, (MPa)^1/2^
HPMC	23.14	365.01	25.18
PVP	4.549	89.07	22.60

**Table 4:** Contributions of the first-order groups, $C_{i}$, and second-order groups, $D_{j}$, to the dispersion partial solubility parameter, $\delta_{d}$, the polar partial solubility parameter, $\delta_{p}$, and the hydrogen-bonding partial solubility parameter, $\delta_{H}$, that appears in HPMC and PVP [33,51].

Polymer/ Structural Groups	Contributions			Occurrences
HPMC				
First-order group	$C_{i,d}$	$C_{i,p}$	$C_{i,H}$	$N_{i}$
–CHO–	111.46	1.6001	0.4873	2
–CH<	82.94	0.6051	−0.2064	3
–CH_2_–	1.82	−0.3141	−0.3877	1
–CH_2_O–	13.4	0.8132	−0.1196	0.27
–OH	−29.97	1.0587	7.3609	1.5
–OCH_3_	−68.07	0.0089	0.2676	0.87
–CH_3_	−123.01	−1.6444	−0.7458	0.27
–CH<	82.94	0.6051	−0.2064	0.27
–OH	−29.97	1.0587	7.3609	0.27
PVP				
First-order group	$C_{i,d}$	$C_{i,p}$	$C_{i,H}$	$N_{i}$
–CH_2_–	1.82	−0.3141	−0.3877	1
–CH<	82.94	0.6051	−0.2064	1
Second-order group	$D_{j,d}$	$D_{j,p}$	$D_{j,H}$	$M_{j}$
N_cyclic_H_m_-C_cyclic_ = O	93.54	2.0813	1.2226	1

**Table 5:** Hansen solubility parameters of HPMC, PVP, and different solvents from literature (MPa)^1/2^.

Sample	Partial Solubility Parameters			
Polymers	$\delta_{d}$	$\delta_{p}$	$\delta_{H}$	$\delta_{t}$
HPMC	19.35	14.13	20.64	31.62
PVP	18.23	9.98	8.33	22.39
Solvents [63]				
Water (W)	15.6	16.0	42.3	47.8
Methanol (Me)	15.1	12.3	22.3	29.6
Acetic acid (AA)	14.5	8.0	13.5	21.4
N,N—Dimethylformamide (DMF)	17.4	13.7	11.3	24.9
N—Methyl—2—pyrrolidone (NMP)	18.0	12.3	7.2	23.0

**Table 6:** Parameters obtained by Hansen theory for systems HPMC/solvents and PVP/solvents, using modeling technique of 3D solubility: solubility parameter distance, $R_{a}$, and relative energy difference, *RED*.

Polymer	Solvent	$R_{a}$	*RED*
HPMC	Water (W)	22.998	6.927
	Methanol (Me)	8.852	2.666
	Acetic acid (AA)	13.515	4.071
	N,N—Dimethylformamide (DMF)	10.131	3.052
	N—Methyl—2—pyrrolidone (NMP)	13.83	4.166
PVP	Water (W)	34.889	2.801
	Methanol (Me)	15.486	1.243
	Acetic acid (AA)	9.297	0.746
	N,N—Dimethylformamide (DMF)	5.04	0.404
	N—Methyl—2—pyrrolidone (NMP)	2.617	0.21

**Table 7:** Viscometric parameters obtained by Huggins and Wolf equations for HPMC/PVP polymers mixture in water at 25 °C.

**Systems**	$\phi_{HPMC}$	${\left[\eta \right]}_{H},(\mathrm{dL}/g)$	${\left[\eta \right]}_{W},(\mathrm{dL}/g)$	$k_{H}$	B
HPMC	1	2.638	3.150	0.519	0.057
75/25	0.75	1.041	1.824	0.654	0.423
50/50	0.5	0.695	0.800	0.725	−0.041
25/75	0.25	0.433	0.463	0.382	0.043
PVP	0	0.203	0.221	0.458	0.165

## Data Availability

Not applicable.
